# Prevalence, Clinical Characteristics, and Treatment of Patients with Resistant Hypertension: A Single-Center Study

**DOI:** 10.3390/jcdd11090279

**Published:** 2024-09-05

**Authors:** Stefan Naydenov, Emil Manov, Nikolay Runev

**Affiliations:** Department of Internal Diseases “Prof. St. Kirkovich”, Medical University of Sofia, 1431 Sofia, Bulgaria; doctor_emil_manov@abv.bg (E.M.); nrunev@abv.bg (N.R.)

**Keywords:** resistant, hypertension, treatment, control, cardiovascular, risk

## Abstract

Background: Resistant hypertension (HTN) is associated with a high risk of cardiovascular complications. Our study aimed to assess the prevalence, characteristics, and treatment of patients with resistant HTN. Methods: We screened 4340 consecutive cardiovascular patients hospitalized in our clinic and identified 3762 with HTN. Of them, 128 fulfilled criteria for resistant HTN and were included in our study. We matched these patients to 128 hospitalized patients with controlled HTN. Results: Resistant HTN patients comprised 3.4% of all hypertensive individuals. Most of these patients (67.2%) were at high or very high cardiovascular risk compared to controlled HTN patients (40.6%); *p* < 0001. Resistant HTN patients more commonly had concomitant chronic kidney disease (CKD) (60.9%), overweight/obesity (52.3%), dyslipidemias (35.2%), smoking (27.3%), and diabetes (21.9%) compared to controlled HTN patients (37.5%, 29.7%, 28.1%, 14.1%, and 7.8%, respectively); *p* < 0.001. Regression analysis showed the strongest association of resistant HTN with CKD (OR 6.64), stage III HTN (OR 3.07), and obesity/overweight (OR 2.60). In contrast, single-pill combinations (SPCs) were associated with a lower likelihood of uncontrolled HTN (OR 0.58). Conclusions: Resistant HTN represented a small proportion of all hypertensives in our study, but it was characterized by high/very high cardiovascular risk. Optimized therapy including increased use of SPCs could improve blood pressure control and long-term prognosis for these patients.

## 1. Introduction

Arterial hypertension (HTN) is defined as a persistent increase in systolic blood pressure (SBP) ≥ 140 mmHg and/or diastolic blood pressure (DBP) ≥ 90 mmHg, as measured repeatedly in clinical settings. When necessary, this diagnosis can be confirmed by a 24-h ambulatory blood pressure (BP) measurement or multiple home BP readings [1,2,3,4]. Today, HTN is a manageable condition for most patients [2,3,4,5]. Modern therapeutic strategies, including single-pill combinations of different pharmacological classes, can achieve target BP levels in approximately 60% of patients on dual therapy and in 90–95% of those on triple therapy [2]. However, some patients remain hypertensive despite optimal therapy, and many of these cases are classified as “Resistant HTN” [2,3,4,6,7]. According to most guidelines, HTN is considered “true resistant” if treatment with optimal or best-tolerated doses of three or more drugs—including a thiazide or thiazide-like diuretic, a blocker of the renin–angiotensin–aldosterone system (RAAS), and a calcium channel blocker (CCB)—plus appropriate lifestyle measures fails to reduce office BP to <140/90 mmHg [1,2,3,4,8].

The prevalence of resistant HTN is estimated to be 5–10% of the hypertensive population in most countries, based on clinical studies and registries (up to 19% according to some publications) [2,3,4,6,9,10,11]. However, the true prevalence of resistant HTN is difficult to estimate due to its dependence on numerous factors, including clinical settings (general population, tertiary referral center, clinical trials), classes and optimal doses of the antihypertensive drugs used, exclusion or retention of non-adherent patients, BP measurement methods, and the definition of target BP values representing BP control [2,11,12,13,14]. The exclusion of secondary HTN and confirmation of good adherence to therapy are also required to define true resistant HTN and exclude pseudo-resistant HTN [1,2,3,4,15].

Resistant hypertension is often found among patients with certain comorbidities and risk factors, such as chronic kidney disease (CKD), type 2 diabetes mellitus (DM), obesity, obstructive sleep apnea (OSA), high sodium intake, sedentary lifestyle, excessive alcohol consumption, and older age [2,6,9,13,16,17]. The pathophysiology of resistant HTN involves an interplay of multiple neurohumoral factors, such as increased sympathetic activity and elevated levels of aldosterone, endothelin-1, and vasopressin [2,6,11,18,19,20,21,22]. These factors contribute to increased peripheral vascular resistance, sodium retention and volume overload, and increased arterial stiffness, leading to cardio-renal damage [2,6,11,14,17,19,21,23].

Patients with resistant HTN are generally considered at higher risk for developing subclinical and clinically manifest hypertension-mediated organ damage (HMOD), such as left ventricular hypertrophy, atherosclerotic vascular disease, atrial fibrillation, heart failure, ischemic and hemorrhagic stroke, CKD (including end-stage renal failure), and premature cardiovascular death [2,11,13,16]. According to some clinical studies, many of these patients have a 10-year risk of a fatal or non-fatal cardiovascular event greater than 20% at the time of diagnosis [2,3,4,6,11].

Unresolved questions regarding the true prevalence of resistant HTN, the clinical characteristics of this heterogeneous group of hypertensive patients, and the therapeutic challenges faced by many clinicians provided the rationale for our clinical study. This study aimed to address some of these issues related to resistant HTN.

## 2. Materials and Methods

We conducted an observational, retrospective, non-interventional study. As a first step, we consecutively screened 4340 patients for HTN. Screening included patients hospitalized at our clinic with various cardiovascular pathologies/diagnoses from 1 July 2018, to 27 March 2024. We identified HTN in 3762 (86.7%) of all patients. In the next step, the hypertensive population was screened for resistant HTN using criteria recommended by the 2018 European Society of Cardiology (ESC)/European Society of Hypertension (ESH) Guidelines and 2023 ESH Guidelines for the management of arterial hypertension [1,2].

Inclusion criteria for our study were as follows: (1) age ≥ 18 years; (2) an established diagnosis of “Arterial hypertension” according to the 2018 ESC/ESH Guidelines and/or 2023 ESH Guidelines for the diagnosis and treatment of this disease; (3) maintenance of office SBP ≥ 140 mmHg and/or office DBP ≥ 90 mmHg after ≥1 month of treatment with optimal or best-tolerated doses of three or more drugs, including a thiazide/thiazide-like diuretic, an RAAS blocker (either an angiotensin-converting enzyme inhibitor (ACEi) or an angiotensin II receptor blocker (ARB)), and a calcium channel blocker (CCB); (4) uncontrolled HTN confirmed by 24-h Holter-BP monitoring; (5) good adherence to the prescribed treatment (≥80% of the treatment period); and (6) patient agreement and signed informed consent at hospital admission to participate in all planned physical, instrumental, and laboratory investigations.

Exclusion criteria for the study were as follows: (1) patients with HTN at target BP values achieved using ≤3 antihypertensive drugs; (2) patients with uncontrolled HTN using ≥3 drugs, but not at optimal doses or not including ACEi/ARB + CCB + thiazide/thiazide-like diuretic or a treatment period of <1 month; (3) an established diagnosis of “secondary HTN”; (4) suspected/confirmed pseudo-resistant HTN; and (5) patient clinical condition and/or comorbid factors/diseases making the planned instrumental investigations unfeasible.

After applying these criteria, we identified 128 patients with resistant HTN, representing 2.9% of all cardiovascular patients (*n* = 4340) screened and 3.4% of the hypertensive population (*n* = 3762) in our study. Of the patients with resistant HTN, 63 (49.2%) were male, and 65 (50.8%) were female (*p* = 0.860). The median age was 58.0 years, with an interquartile range (IQR) of 46.0–69.0 years.

In the third step, we used propensity score matching to match 128 hospitalized hypertensive patients who had achieved target BP values (controlled HTN) to those with resistant HTN. These patients had to meet the following inclusion criteria: (1) office SBP 130–139 mmHg for patients aged ≥ 65 years and 120–129 mmHg for those aged < 65 years; (2) office DBP 70–80 mmHg; (3) mean 24-h Holter-monitoring values <130 mmHg for SBP and <80 mmHg for DBP; (4) mean daytime Holter-monitoring SBP <135 mmHg and <85 mmHg for DBP; (5) mean nighttime Holter-monitoring SBP < 120 mmHg for SBP and <70 mmHg for DBP; (6) control of HTN achieved by ≤3 antihypertensive drugs (with a free or a single-pill combination if >1 drug used) from different classes at standard or maximal doses taken for at least 4 weeks; and (7) patient agreement and signed informed consent at hospital admission to participate in all planned physical, instrumental, and laboratory investigations.

**Figure 1:**
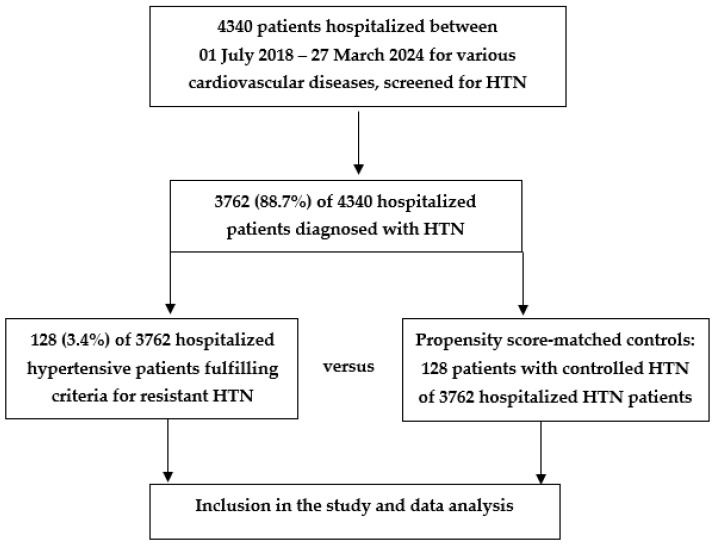
Screening and selection of participants for our study. HTN—arterial hypertension.

Figure 1 summarizes the participant inclusion process for our study.

The study was conducted following the ethical standards outlined in the 1964 Declaration of Helsinki and its later amendments, guidelines for good clinical practice, and local regulations. Approval by a local ethics committee was not required for this type of clinical study/scientific research (observational, retrospective, non-interventional) in our country. All participants provided signed informed consent at hospital admission, agreeing to be examined and treated according to the diagnostic and treatment plan proposed by the clinician/hospital team, and that their results could be used anonymously for scientific purposes. The study was registered at https://www.clinicaltrials.gov (accessed on 7 February 2024) with reference number KPVB0001RH.

Patient information was collected in a structured questionnaire form that included demographic characteristics, medical history (complaints, cardiovascular risk factors, comorbidities, treatment, etc.), and clinical, instrumental, and laboratory findings of interest. Data were gathered directly from the medical records of the patient’s hospitalization and other available documents. Instrumental investigations included electrocardiography (ECG), transthoracic echocardiography, 24-h Holter BP monitoring, and routine laboratory parameters. Contrast computed tomography (CT) imaging of the kidneys with renovasography was performed on all patients with resistant HTN and on patients with controlled HTN whose kidney echography and duplex Doppler sonography showed abnormal findings.

Patient cardiovascular risk was calculated according to the 2021 ESC Guidelines on cardiovascular prevention and the 2023 ESH Guidelines for the management of HTN [2,24]. In apparently healthy individuals under 70 years of age without established atherosclerotic cardiovascular disease (ASCVD), DM, CKD, genetic/rare lipid, or BP disorders, a 10-year fatal and non-fatal cardiovascular risk estimation was performed using the SCORE2 chart. This chart accounts for age, gender, smoking status, total cholesterol, and BP level and is calibrated to the country of residence. For individuals with ASCVD, DM, CKD, or genetic/rare lipid or BP disorders, cardiovascular risk was calculated using risk modifiers and the three cardiovascular risk categories recommended by the 2021 ESC Guidelines [2,24].

### Statistical Analysis

For data processing and statistical analysis, we used IBM SPSS Statistics 19.0 software (SPSS Inc., Chicago, IL, USA). Categorical variables were expressed as absolute numbers (percentage, %), and differences were evaluated using the chi-square test. Normally distributed continuous variables were expressed as means ± standard deviation, and comparisons were made using the *t*-test for independent samples or analysis of variance. Non-normally distributed continuous variables were expressed as median (interquartile range) and compared using the Mann–Whitney U test or the Kruskal–Wallis H test. Association between the independent variables (concomitant conditions/factors/diseases) and the dependent variables (resistant/controlled HTN) was determined by logistic regression analysis with the strength of each variable demonstrated by the odds ratio (OR). A *p*-value < 0.05 was considered statistically significant.

## 3. Results

### 3.1. Patient Demographic and Clinical Characteristics

Table 1 presents the demographic characteristics, concomitant risk factors, comorbidities, and cardiovascular risk profiles of the participants in our study. Both genders were almost equally represented in the overall study population, as well as in the two groups compared (resistant HTN vs. controlled HTN). Patients with resistant HTN were younger, with a median age difference of 5 years compared to those with controlled HTN. As shown in Table 1, both groups had a comparable duration of elevated blood pressure (BP); however, patients with resistant HTN more frequently presented with moderate and severe hypertension (systolic BP ≥ 160 mmHg and/or diastolic BP ≥ 100 mmHg). Subclinical and clinically manifested hypertensive-mediated organ damage (HMOD) was also more common among patients with resistant HTN, indicating a more advanced stage of hypertension.

Risk factors and comorbidities that were more prevalent among patients with resistant HTN included active smoking, overweight/obesity, type 2 diabetes mellitus (DM), and chronic kidney disease (CKD). Ischemic heart disease (IHD) showed borderline statistical significance, while other risk factors and comorbidities had comparable prevalence in both groups.

### 3.2. Laboratory and Instrumental Investigations

Table 2 displays the basic laboratory parameters of the study participants. Patients with resistant HTN had higher levels of fasting blood glucose and creatinine and a lower estimated glomerular filtration rate (eGFR), as calculated using the equation recommended by the 2021 Guidelines of the CKD Epidemiology Collaboration (CKD-EPI) group, compared to patients with controlled HTN [25]. For all other laboratory parameters, both groups were comparable.

**Figure 2:**
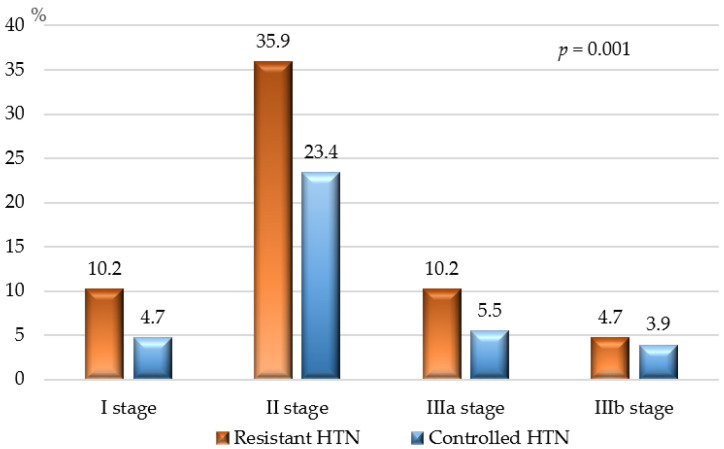
CKD stage of patients with resistant and controlled HTN according to the equation recommended by the 2021 Guidelines of CKD Epidemiology Collaboration Group [25]; *p* — *p*-value for statistical significance.

Figure 2 shows CKD stages based on eGFR calculated using the 2021 CKD-EPI equation. Patients with resistant HTN had more advanced CKD compared to those with controlled HTN; however, in both groups, the severity of kidney dysfunction ranged from moderate to severe (Stage IIIb) at worst. No patients in either group were classified as having severe (Stage IV) or terminal (Stage V) CKD.

Table 3 provides the office BP values, 24-h Holter BP monitoring values, and office-measured heart rate (HR) for the study population. Patients with resistant HTN had significantly higher BP values in both office and Holter BP measurements compared to patients with controlled HTN. The pulse pressure (the difference between systolic and diastolic blood pressure), an important risk factor for cardiovascular events—particularly strokes—was also significantly higher in patients with resistant HTN compared to those with controlled HTN. No statistically significant difference in HR was found between the two groups.

**Figure 3:**
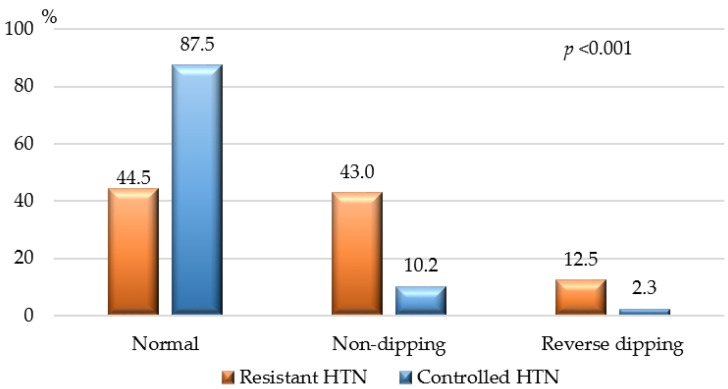
Dipping state of patients with resistant and controlled HTN. Normal dipping state—10–20% decrease in the nighttime SBP and DBP to the daytime values; non-dipping—1–9% decrease in nighttime SBP and/or DBP compared to the daytime values; reverse dipping—nighttime SBP and/or DBP increase compared to the daytime values; *p*—*p*-value for statistical significance.

Figure 3 shows the BP dipping status of the study population, assessed by 24-h Holter-BP monitoring. According to our results non-dipping and reverse dipping state were significantly more common among patients with resistant HTN.

### 3.3. Risk Profile of the Study Population

**Figure 4:**
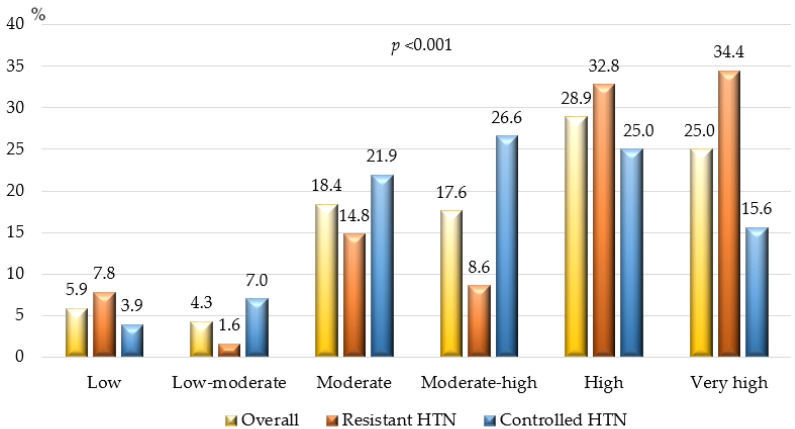
Cardiovascular risk of the study population. HTN—arterial hypertension; *p* — *p*-value for statistical significance.

Figure 4 illustrates the cardiovascular risk profiles of the study participants. Risk was calculated according to the algorithms proposed by the 2021 ESC Guidelines on cardiovascular disease prevention in clinical practice and the 2023 ESH Guidelines for the management of HTN. Results showed that a significant proportion of all hypertensive patients in our study (~54%) were at high or very high cardiovascular risk: >67% of patients with resistant HTN and >40% of those with controlled HTN, *p* <0.001.

### 3.4. Treatment of the Study Population

Table 4 outlines the classes of antihypertensive drugs used by the patients prior to re-evaluation of their therapeutic approach. Calcium channel blockers, particularly dihydropyridine-type (DHP-CCB), diuretics (mainly thiazide/thiazide-like), and angiotensin receptor blockers (ARBs) were the most frequently prescribed drug classes for patients with resistant HTN. The same classes were preferred for patients with controlled HTN, but prescription rates were significantly lower compared to those with resistant HTN. The use of second-line antihypertensive drugs, including mineralocorticoid receptor antagonists (MRAs), α1-receptor blockers, and centrally acting agents, was higher in patients with resistant HTN.

Regarding the number of antihypertensive classes prescribed, the median was 3 (IQR 3–5) for patients with resistant HTN and 2 (IQR 2–3) for those with controlled HTN, *p* <0.001. The median number of antihypertensive tablets taken daily was 4 (IQR 3–6) for patients with resistant HTN and 1 ½ (IQR 1–3) tablets for those with controlled HTN, *p* <0.001.

**Figure 5:**
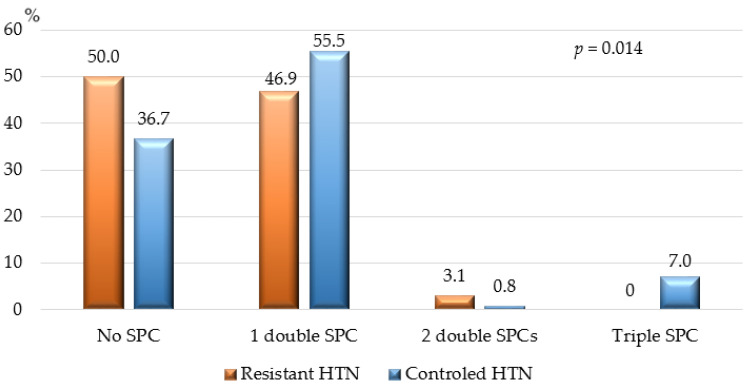
Treatment with double and triple single-pill combinations. HTN—arterial hypertension; SPC—single-pill combination; *p*—*p*-value for statistical significance.

Single-pill combinations (SPCs) containing 2–3 antihypertensive classes were used by 145 patients (56.6%): 64 patients (50.0%) with resistant HTN and 81 patients (63.3%) with controlled HTN, *p* = 0.002. Figure 5 shows the percentage of patients with resistant versus controlled HTN treated with double and triple SPCs.

### 3.5. Impact of Concomitant Risk Factors/Diseases and Drug Treatment on HTN Control

Table 5 presents the variables (demographic factors, comorbidities, and treatment regimens) most strongly associated with resistant HTN in our study. We found a significant positive association between resistant HTN and CKD, advanced stages of hypertension, obesity/overweight, concomitant IHD, type 2 DM, and active smoking. Other analyzed factors, such as gender, age, dyslipidemia, cerebrovascular disease, peripheral artery disease (PAD), atrial fibrillation (AF), heart failure (HF), and others, did not significantly influence the odds ratio for resistant HTN. Treatment with single-pill combinations and certain drug classes was associated with a higher likelihood of achieving controlled HTN.

## 4. Discussion

In our study, patients with resistant hypertension (HTN) accounted for 3.4% of all hypertensive patients—a prevalence slightly lower than but generally comparable to that reported by other authors [1,2,3,4,8,9,11]. According to the 2018 ESC/ESH and 2023 ESH Guidelines, patients with true resistant HTN constitute approximately 5% of all individuals with high BP [1,2]. While the percentage of resistant HTN patients in our study and other studies may not seem significant at first glance, considering the global hypertensive population—approximately 1.28 billion adults aged 30–79 years—it translates to over 43 million people with HTN resistant to treatment, constantly exposed to high and very high cardiovascular risk [9,10,16,17,26,27].

It is important to point out that the definition of resistant HTN, adopted by many guidelines, is based on the persistence of office BP values ≥140/90 mmHg despite appropriate lifestyle measures and treatment with optimal or best-tolerated doses of at least three drugs (a thiazide/thiazide-like diuretic, a RAAS blocker, and a CCB) [1,2,3,6,8]. However, these proposed cut-off BP values are inconsistent with the target BP values of <130/80 mmHg recommended for many hypertensive patients [1,2,3,4,8]. For this reason, some authors suggest lower office BP values (<130/80 mmHg) for defining resistant HTN or values based on the target BP for the specific hypertensive population, a viewpoint we fully support [4,11,14,15,27]. Such an amendment to the criteria could significantly increase the percentage of patients with true resistant HTN [2,4,11,13,14,15].

In our study, the assessment of concomitant risk factors and diseases revealed that 60% of patients with resistant HTN had CKD. Other studies demonstrate that 60–80% of patients with a kidney disease have high BP [2,6,11,16,27,28]. Our regression analysis demonstrated that CKD was most strongly associated with difficult-to-control HTN among all factors analyzed, increasing the likelihood of resistant HTN 6.6-fold, significantly higher than the 2–3-fold increase reported by other authors [2,6,11,28]. Ten of our patients with resistant HTN had concomitant atherosclerotic renal artery disease. While some authors may consider them as patients with secondary HTN, we regard these cases as primary HTN with superimposed renovascular disease [2,4,6,7,11,15,27]. According to their medical records, at the time of HTN diagnosis ≥10–15 years ago, there was no evidence of concomitant renovascular or renal parenchymal disease based on medical history, clinical, and instrumental investigations. Additionally, these patients had well-controlled HTN for years before the development of stenotic atherosclerotic plaques in the renal arteries, which subsequently worsened hypertension. The superimposed atherosclerotic renovascular disease complicated the control of primary HTN, rendering it resistant to treatment. However, we do not consider this as true secondary HTN, acknowledging that this is a “grey zone” in HTN classification (primary/secondary).

Other factors in our patient population strongly associated with resistant HTN included overweight/obesity, insulin resistance/type 2 diabetes, which increased the odds of uncontrolled HTN by approximately 2.6-, 2.1-, and 1.9-fold, respectively. According to other authors, diabetes increases the likelihood of resistant HTN by about 2-fold, and obesity by 2- to 4.5-fold [6,11,29,30,31]. Most publications we reviewed mentioned that smoking was more common among patients with resistant HTN, but few provided details on the level of association [2,14,30,32].

In our study, ischemic heart disease (IHD), as well as stage II and stage III HTN, were also strongly associated with resistant HTN. Smith et al. reported that 38% of their patients with coronary artery disease had resistant HTN [22]. These patients had also much worse cardiovascular outcomes compared to those with controlled HTN [22]. In our view, IHD and the medications used to treat it are unlikely to directly cause resistance to HTN treatment. The connection between these two conditions likely involves the increased activity of the renin–angiotensin–aldosterone and sympathetic nervous systems, leading to vasoconstriction, increased arterial resistance, endothelial damage, and vascular remodeling [7,19,23,33,34,35]. Some of these processes may also contribute to the impaired blood pressure dipping state observed in HTN patients [6,17,36]. In our study, non-dipping and reverse BP dipping were significantly more common among patients with resistant HTN (about 53%) compared to those with controlled HTN (approximately 12%). Ingabire et al. reported a non-dipping BP pattern in up to 78% of their patients with uncontrolled HTN [37].

We did not find statistically significant sex differences between resistant and controlled HTN patients. In some studies, resistant HTN was more prevalent among males [9,16,17,38,39]. Interestingly, our patients with resistant HTN were younger than those with controlled HTN, despite our expectation of the opposite. Vascular aging, characterized by the loss of elastic fibers and arterial stiffening, is considered one of the pathogenic mechanisms leading to high BP [6,17,21]. In other studies, the incidence and prevalence of resistant HTN increased with age [9,13,16,17,30].

An important finding from our study was the relatively low percentage of patients with resistant HTN treated with single-pill combinations (SPCs) (~57%), which could be at least partially responsible for insufficient HTN control. Strong evidence from clinical trials suggests that SPCs significantly improve treatment adherence and BP control [2,40,41]. According to the 2023 ESH Guidelines on HTN, double combinations are expected to achieve sufficient BP control in approximately 60%, and triple SPCs in about 90% of all hypertensive patients [2]. Our results showed that patients treated with SPCs had a 42% lower odds of resistant HTN compared to those on free combinations after adjusting for other influencing factors. We did not find clinical data from other studies providing exact numbers on how SPCs reduce the risk or odds ratio (OR) for developing HTN and/or improving BP control in resistant HTN patients compared to free combinations.

In our study, more than 50% of hypertensive patients were at high or very high cardiovascular risk, particularly those with resistant HTN (~67%). Epidemiological data from other authors show variable levels of cardiovascular risk, depending on the hypertensive populations analyzed and the concomitant risk factors or diseases [2,6,9,17]. However, most agree that patients with resistant HTN should be regarded as being at high or very high cardiovascular risk [2,12,16,17]. This should be considered in a holistic therapeutic approach that necessitates sufficient control of all concomitant factors and diseases (obesity/overweight, dyslipidemia, diabetes mellitus/impaired glucose tolerance, smoking, etc.) [2,3,11,24]. After all, BP reduction should not be an end in itself but rather part of a global therapeutic strategy aimed at lowering total cardiovascular risk [2,3,24].

### Study Limitations

Our study has several limitations. First, the number of patients we included does not allow for the extrapolation of our results to the entire population of patients with resistant HTN. Second, we analyzed the prevalence and influence of some, but not all factors and diseases associated with the development of resistant HTN. Third, our study included only hospitalized hypertensive patients; ambulatory patients may have a different profile. Fourth, the results and analysis represent a momentary “snapshot” of the situation because we conducted a retrospective, cross-sectional study; results after treatment optimization were not available for analysis.

## 5. Conclusions

Patients with resistant HTN represented a relatively small proportion of all hypertensive patients in our study, but they were characterized by high or very high cardiovascular risk. The factors most strongly associated with resistant HTN were CKD, Stage II and III HTN, obesity/overweight, IHD, Type 2 diabetes mellitus, and active smoking, whereas treatment with SPCs was associated with a lower likelihood of uncontrolled BP. The therapeutic strategy for patients with resistant HTN should be holistic, aiming to achieve control not only of HTN but also of all concomitant risk factors and diseases, as the ultimate goal is to reduce total cardiovascular risk.

## Figures and Tables

**Table 1:** Patient characteristics at inclusion in the study.

Characteristics	Total *n* = 256	Resistant HTN *n* = 128	Controlled HTN *n* = 128	*p*
Age (years), median (IQR)	61.0 (51.0–69.0)	58.0 (46.0–69.0)	63.0 (57.0–68.8)	0.003
Gender, *n* (%)				
Males	130 (50.8%)	63 (49.2%)	67 (52.3%)	0.354 ^#^
Females	126 (49.2%)	65 (50.8%)	61 (47.7%)	
HTN grade, *n* (%)				
Mild	74 (28.9%)	3 (2.3%)	71 (55.5%)	<0.001 ^#^
Moderate	88 (34.4%)	53 (41.4%)	35 (27.3%)	
Severe	94 (36.7%)	72 (56.3%)	22 (17.2%)	
HTN stage, *n* (%)				
Ist stage	79 (30.9%)	32 (25.0%)	47 (36.7%)	0.001 ^#^
IInd stage	109 (42.6%)	50 (39.1%)	59 (46.1%)	
IIIrd stage	68 (26.6%)	46 (35.9%)	22 (17.2%)	
HTN duration (years), median (IQR)	10 (5.3–18)	10 (5.3–20)	10 (5.3–16)	0.555
Smoking, *n* (%)				
Active	53 (20.7%)	35 (27.3%)	18 (14.1%)	0.030
Ex-smoker	16 (6.3%)	8 (6.3%)	8 (6.3%)	1.000
Alcohol consumption *	33 (12.9%)	19 (14.8%)	14 (10.9%)	0.456
Overweight/obesity, *n* (%)	105 (41.0%)	67 (52.3%)	38 (29.7%)	0.001
Dyslipidemia, *n* (%)	81 (31.6%)	45 (35.2%)	36 (28.1%)	0.282
Type 2 diabetes, *n* (%)	38 (14.8%)	28 (21.9%)	10 (7.8%)	0.034
IGT, *n* (%)	20 (7.8%)	9 (7.0%)	11 (8.6%)	0.534
CKD, *n* (%)	126 (49.2%)	78 (60.9%)	48 (37.5%)	0.005
Ischemic heart disease, *n* (%)	31 (12.1%)	21 (16.4%)	10 (7.8%)	0.054
Post-myocardial infarction, *n* (%)	6 (2.3%)	4 (3.1%)	2 (1.6%)	0.342
Post-stroke, *n* (%)	11 (4.3%)	8 (6.3%)	3 (2.3%)	0.263
TIA, *n* (%)	3 (1.2%)	1 (0.8%)	2 (1.6%)	0.283
PAD, *n* (%)	11 (5.5%)	7 (8.3%)	4 (3.5%)	0.208
Heart failure, *n* (%)	38 (14.9%)	23 (18.0%)	15 (11.8%)	0.132

CKD—chronic kidney disease; HTN—arterial hypertension; IGT—impaired glucose tolerance; IQR—interquartile range; Mild HTN—SBP 140–159 and/or DBP 90–99 mmHg; Moderate HTN—SBP 160–179 and/or DBP 100–109 mmHg; Severe HTN—SBP ≥180 and/or DBP ≥180 mmHg; PAD—peripheral arterial disease; TIA—transitory ischemic attack; * ≥14 units per week for males and ≥8 units per unit for females (1 unit = 25 mL of standard alcohol drink with 40.0% alcohol content or 125 mL of wine with 12.0% alcohol content or 250 mL beer with 5.0% alcohol content); ^#^—the comparison is between the groups (resistant versus controlled HTN); *p*—*p*-value for statistical significance.

**Table 2:** Basic laboratory parameters of the study population.

Parameter	Total	Resistant HTN	Controlled HTN	*p*
Potassium, mmol/L, median (IQR)	4.6 (4.2–4.9)	4.6 (4.3–4.9)	4.6 (4.2–4.9)	0.351
Sodium, mmol/L, median (IQR)	142 (139–145)	142 (139–145)	142 (140–145)	0.546
Hemoglobin, g/L, median (IQR)	148 (136–158)	147 (135–159)	148 (137–157)	0.606
Hematocrit, L/L, median (IQR)	0.44 (0.42–0.46)	0.44 (0.42–0.47)	0.44 (0.42–0.46)	0.126
Fasting glucose, mmol/L, median (IQR)	5.3 (4.8–6.2)	5.6 (4.9–6.3)	5.3 (4.8–5.9)	0.048
Creatinine, µmol/L, median (IQR)	76 (66–92)	79 (69–96)	73 (65–84)	0.007
eGFR, mL/min/1.73 m^2^, median (IQR)	92 (75–101)	88 (71–101)	94 (65–97)	0.042

eGFR—estimated glomerular filtration rate (according to the equation recommended by the 2021 Guidelines of CKD Epidemiology Collaboration Group) [25]; IQR—interquartile range; *p*—*p*-value for statistical significance.

**Table 3:** Office BP measurement with pulse pressure calculation, 24-h Holter-BP monitoring, and office-measured HR.

Characteristics	Total *n* = 256	Resistant HTN *n* = 128	Controlled HTN *n* = 128	*p*
Office SBP (mm Hg), median (IQR)	139.0 (130.0–150.0)	150.0 (145.0–160.0)	130.0 (122.0–134.8)	<0.001
Office DBP (in mm Hg), median (IQR)	85.0 (80.0–90.0)	90.0 (90.0–97.0)	80.0 (77.0–82.0)	<0.001
Pulse pressure (in mm Hg), median (IQR)	51.0 (50.0–60.0)	60.0 (50.0–70.0)	50.0 (45.0–53.0)	<0.001
Daytime Holter-monitoring SBP (in mm Hg), median (IQR)	134.0 (128.5–145.0)	145.0 (139.0–152.0)	128.5 (123.0–132.0)	<0.001
Daytime Holter-monitoring DBP (in mm Hg), median (IQR)	84.0 (78.0–90.0)	90.0 (88.0–95.0)	78.0 (72.0–82.0)	<0.001
Nighttime Holter-monitoring SBP (in mm Hg), median (IQR)	123.0 (115.0–137.0)	137.0 (130.0–145.0)	115.0 (111.0–118.0)	<0.001
Nighttime Holter-monitoring DBP (in mm Hg), median (IQR)	74.0 (65.0–85.5)	86.0 (79.0–92.0)	65.0 (62.3.5–68.0)	<0.001
24 h Holter-monitoring SBP (in mm Hg), median (IQR)	132.0 (122.0–141.0)	141.0 (137.0–147.0)	122.0 (118.0–126.0)	<0.001
24 h Holter-monitoring DBP (in mm Hg), median (IQR)	79.0 (71.0–88.0)	88.0 (84.0–93.0)	71.0 (68.0–75.0)	<0.001
Heart rate (beats/min.), median (IQR)	74 (67–80)	74 (65–80)	75 (68.0–80.0)	0.406

DBP—diastolic blood pressure; HR—heart rate; IQR—interquartile range; SBP—systolic blood pressure; *p*—*p*-value for statistical significance.

**Table 4:** Antihypertensive classes used by the patients, included in our study.

Antihypertensive Classes	Total *n* = 256	Resistant HTN *n* = 128	Controlled HTN *n* = 128	*p*
ACEi, *n* (%)	78 (30.5%)	34 (26.6%)	44 (34.4%)	0.222
ARB, *n* (%)	147 (57.4%)	82 (64.1%)	65 (50.8%)	0.037
CCB, *n* (%)	181 (70.7%)	107 (83.6%)	74 (57.8%)	<0.001
DHP-CCB	177 (69.1%)	104 (81.3%)	73 (57.0%)	<0.001
Non-DHP-CCB	4 (1.6%)	3 (2.3%)	1 (0.8%)	<0.001
Diuretics, *n* (%)	183 (71.5%)	106 (82.8%)	77 (60.2%)	<0.001
Thiazide/thiazide-like	120 (46.9%)	63 (49.2%)	57 (44.5%)	<0.001
Loop	50 (19.5%)	32 (25.0%)	18 (14.1%)	<0.001
Thiazide + Loop	13 (5.1%)	11 (8.6%)	2 (1.6%)	<0.001
Beta-blockers, *n* (%)	125 (48.8%)	76 (59.4%)	49 (38.3%)	0.001
MRA, *n* (%)	27 (10.5%)	21 (16.4%)	6 (4.7%)	0.002
α1-receptor blockers, *n* (%)	17 (6.6%)	15 (11.7%)	2 (1.6%)	0.001
Centrally acting agents, *n* (%)	59 (23.0%)	47 (36.7%)	12 (9.4%)	<0.001

ACEi—angiotensin-converting enzyme inhibitor; ARB—angiotensin II receptor blockers; CCB—calcium channel blockers; DHP-CCB—dihydropyridine calcium channel blocker; HTN—arterial hypertension; MRA—mineralocorticoid receptor antagonists; non-DHP-CCB—non-dihydropyridine calcium channel blocker; *p*—*p*-value for statistical significance.

**Table 5:** Association of different factors with resistant HTN.

Variable	OR	95% CI for OR		
		Lower Limit	Upper Limit	*p*
Risk factors/concomitant diseases				
Active smoking	1.944	0.626	6.037	0.033
Type 2 DM	2.072	1.132	790	0.018
IHD	2.316	1.044	5.140	0.039
Stage II HTN	2.467	1.311	4.644	0.005
Obesity/overweight	2.601	1.556	4.349	<0.001
Stage III HTN	3.071	1.559	6.051	0.001
CKD	6.642	3.779	11.674	<0.001
Treatment				
SPC	0.580	0.352	0.956	0.033
ARB	0.799	0.645	0.989	0.038
Diuretics	0.726	0.618	0.854	<0.001
CCB	0.692	0.585	0.817	<0.001
Beta-blockers	0.645	0.496	0.838	0.001
MRA	0.286	0.119	0.684	0.004
Centrally acting agents	0.255	0.142	0.458	0.005
α-1 receptor blockers	0.133	0.031	0.571	<0.001

Logistic regression analysis was used to assess the association of various variables with resistant HTN. Prior independent χ2-test was applied to identify categorical variables with statistically significant relationship with resistant HTN. These variables were entered into the univariate logistic regression model; ARB—angiotensin II receptor blocker; CCB—calcium channel blocker; CI—confidence interval; CKD—chronic kidney disease; DM—diabetes mellitus; IHD—ischemic heart disease; MRA—mineralocorticoid receptor antagonist; HTN—arterial hypertension; OR—odds ratio; *p*—*p*-value for statistical significance; SPC—single-pill combination.

## Data Availability

Data supporting reported results are available and can be provided by Stefan Naydenov, MD, PhD; email: snaydenov@gmail.com.
